# A Comparison of the Physicochemical Properties and Fatty Acid Composition of Indaiá (*Attalea dubia*) and Babassu (*Orbignya phalerata*) Oils

**DOI:** 10.1100/2012/532374

**Published:** 2012-04-19

**Authors:** Bianca Silva Ferreira, Lara Pereira Faza, Mireille Le Hyaric

**Affiliations:** Departamento de Química, Instituto de Ciências Exatas, Universidade Federal de Juiz de Fora, Rua José Lourenço Kelmer, s/n—Campus Universitário, Bairro Martelos, 36036-900 Juiz de Fora, MG, Brazil

## Abstract

The physicochemical properties and fatty acid composition of *Attalea dubia (Mart.) * Burret (indaiá) seed oil were investigated. The oil was extracted in a soxhlet apparatus using petroleum ether and evaluated for iodine, acid, peroxide, ester, and saponification values. The oil was also analyzed using infrared and nuclear magnetic resonance spectroscopy. The fatty acid profile of the oil was determined by GC-MS. For each analysis indaiá oil was compared to *Orbignya phalerata* (babassu) oil. The two oils appeared to be very similar in their fatty acid composition, in which lauric acid (the most abundant), myristic acid, caprylic acid, and capric acid were the four main fatty acids detected. The unsaturated fatty acids content was lower for indaiá oil (5.8%) than for babassu oil (9.4%). The results suggest that indaiá palm tree could be cultivated as a new source of vegetable oil with potential for food and cosmetic industries.

## 1. Introduction

Oils extracted from plants are important not only for their application in food, but also for their industrial applications, such as the production of biodiesel, pharmaceuticals, cosmetics, paints, and others. *Attalea dubia (Mart.) *Burret, also known as Bacuau palm or indaiá, is a palm tree of the *Attalea* species (Arecaceae) [1] endemic to the atlantic rainforest of Brazil. The tree can reach 20 meters high, with a 20–35 cm diameter. Its exploration for the extraction of edible palm heart is mainly illegal [2], as this tree is not a cultivated palm and has been forbidden during the last years in several states of Brazil, due to the importance of indaiá in the alimentation of wild animals [3]. The numerous bark oblong fruits (approximately 6 cm) which look like small coconuts and remind babassu kernels (*Orbignya phalerata*) contain one or two seeds with a white oily endosperm. The oil, produced in local communities from the almonds, can be used to cook.

The physicochemical characterization of the oil, obtained from the seeds of *Attalea dubia *to the best of our knowledge, has not been reported yet. The objective of this work was the determination of the physicochemical properties, NMR spectra and fatty acid profiles of seed oil extracted from *Attalea dubia*, and their comparison with babassu oil, a common vegetable oil used in Brazil [4].

## 2. Materials and Methods

### 2.1. Seed Material

Mature seeds, collected in January 2011 from one palm tree in Itamarati de Minas (Brazil), were handpicked to eliminate the damaged ones. The hard shell was broken, and the seeds were manually ground.

### 2.2. Lipid Extraction

Oil was extracted from the ground seeds in a soxhlet apparatus with petroleum ether for 4 hours. After evaporation of the solvent a yellowish limpid oil was obtained (yield 15%). Babassu oil was purchased form a local market (Januário, Brazil).

### 2.3. Physicochemical Analysis

The following physicochemical properties of oils were determined: density, saponification value, iodine value, peroxide value, acid values, and ester value (content, according to AOCS official methods [5]). All analysis were performed in triplicate.

### 2.4. Fatty Acid Profile

The fatty acid profile was determined as fatty acid methyl esters by gas chromatography-mass spectroscopy. The methyl esters were prepared using the AOCS method [6]. The separation of fatty acid esters was performed on a Shimadzu Gas Chromatograph GC-2010 with a Restek RTX-2330 capillary column (60 m × 0.25 mm × 0.2 mm). The column temperature was programmed at 130°C for 10 min, then increased to 230°C at 5°C/min with a final isothermal period of 13 min. Hydrogen was used as carrier gas with constant linear velocity of 25 cm/sec^−1^. The injector temperature was set at 250°C, with a split ratio of 1 : 10. The flame ionization detector temperature was 250°C. Fatty acid methyl esters (FAMEs) were identified by comparison of retention times with authentic standards (Supelco 37 comp. FAME mix 10 mg/mL in CH2Cl2), and quantification was performed by the internal normalization method.

### 2.5. NMR Analysis

^1^H spectra were acquired from a Bruker Advance DRX 300 MHz, using deuterated chloroform as solvent and trimethylsilane as internal reference [7, 8].

### 2.6. Infrared Spectrometry

FT-IR spectra of the lipid fraction of indaiá and babassu pulp were obtained on a Bomem FT IR MB- 102 spectrometer, with the samples in KBr pellets. The spectra were recorded in the range of 400–3500 cm^−1^ [9].

## 3. Results and Discussion

Indaiá oil was recovered as a yellowish liquid which solidifies when refrigerated.

### 3.1. Physicochemical Analysis

Usual analysis of the oil such as density, saponification value, iodine value, peroxide value, acid values, ester value, and free acid content were realized and compared to the data obtained for babassu oil [10] (Table 1).

The high saponification values of the two oils indicates a high content o triacylglycerol, consistent with the high ester value (>99%). No peroxide was detected in the samples, which means that they can be considered safe for to be consumed [11]. The iodine value was lower for indaiá oil than for babassu oil, suggesting that the first has a higher content of saturated fatty acid. The high saponification value indicates that Indaiá oil has potential to be used in the cosmetic industry [12]. The low acid values determined for both oils indicates that the triacylglycerols have not been hydrolyzed, which could indicate a good stability.

### 3.2. NMR Analysis

**Figure 1:**
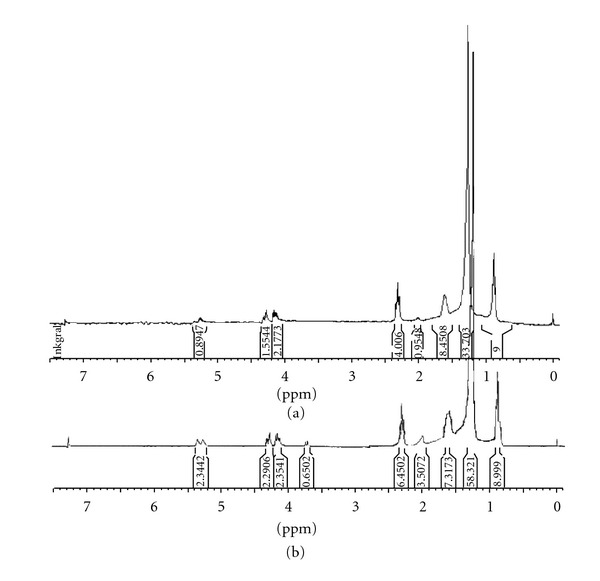
^1^H NMR spectra of indaiá oil (a) and of babassu oil (b) in CDCl_3_.

^1^H NMR spectra of both oils are illustrated in Figure 1, and their respective data are shown in Table 2.

The NMR profiles of the two oils (Table 2) were similar. The main differences were observed comparing the areas of the signals at ~2 ppm and at ~5.3 ppm, corresponding respectively to the methylene an olefin hydrogens of unsaturated fatty acids and to the hydrogens at the unsaturation. Calibrating the signals of terminal methyl groups to 9, which would correspond to a total esterification of the glycerol, we can compare the degree of unsaturations of the two oils. The areas found at 2 ppm were 1 (indaiá) and 3.5 (babassu), and the values found at 5.3 ppm were 0.2 (indaiá) and 0.4 (babassu). These results are consistent with the higher iodine value of the more unsaturated babassu oil.

### 3.3. Fatty Acid Profile

**Figure 2:**
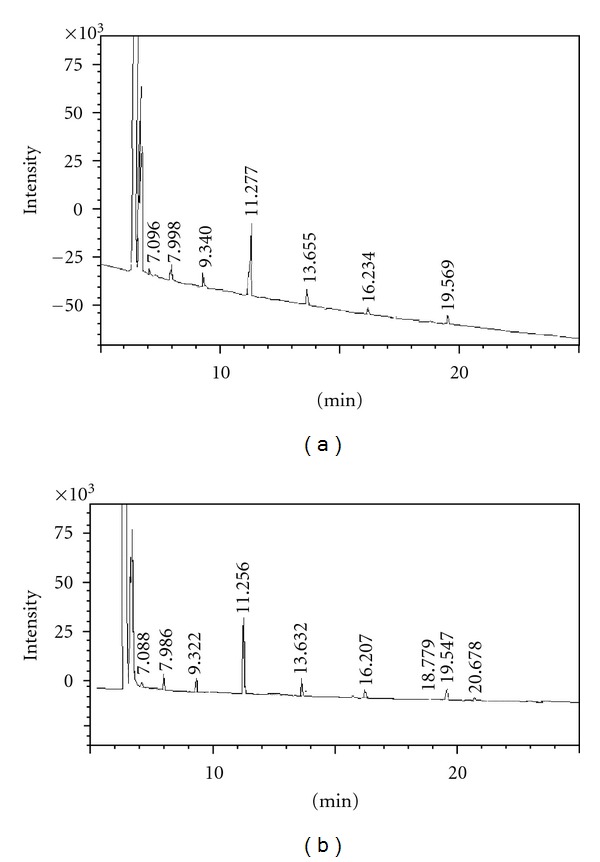
Fatty acid profiles of indaiá (a) and babassu (b) oils.

Gas chromatography spectra of both oils are illustrated in Figure 2, and their respective fatty acid composition is detailed in Table 3.

The main fatty acids present in the two oils were lauric acid (the most abundant, representing more than 50% of the fatty acids detected in the samples), myristic acid (11%), caprylic acid (~10%), and capric acid (8.5% and 9.6%). Total unsaturated fatty acids represent 5.8% of indaiá oil and 9.45% of babassu oil, which is consistent with the iodine values and with the NMR spectra of the oils. 

### 3.4. FT-IR Analysis

**Figure 3:**
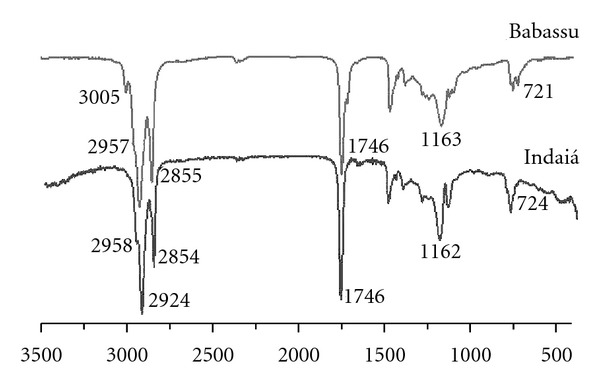
FT-IR spectra of indaiá and babassu oils.

FT-IR spectra of indaiá and babassu oils are illustrated in Figure 3. The assignments of their respective vibrational bands are detailed in Table 4. 

The spectra were almost identical (Figure 3), showing signals in the five main regions used for the analysis of oils and fats: (1) 3100–2805 cm^−1^, (2) 1770–1615 cm^−1^, (3) 1500–1420 cm^−1^, (4) 1345–1230 cm^−1^, and (5) 1150–850 cm^−1^. The unsaturated C–H stretching band was not detected on the infrared spectra of indaiá oil but was observed on the spectra of babassu oil. As expected no band of hydroperoxide group was observed near 3444 cm^−1^, confirming that none of the oils suffered oxidation [13]. The band corresponding to the triglyceride ester groups appears at 1746 cm^−1^, and the bands related to the stretching vibration of the C–O bonding of the ester groups are observed near 1235 and 1160 cm^−1^. 

## 4. Conclusion

The analysis of the indaiá seed oil performed in this work showed its high content of saturated fatty acids, lauric acid being the main one (55.8%). Indaiá oil appeared to be very similar to babassu oil but contains less unsaturated fatty acids than this one (5.8 and 9.45%, resp.). Indaiá oil could be cultivated as a new source of vegetable oil with potential for food and cosmetic industries, as is babassu oil. 

## Figures and Tables

**Table 1:** Physicochemical analysis of indaiá and babassu oils.

	Indaiá	Babassu
Density (g/cm^3^)	0.917 ± 0.003	0.920 ± 0.002
Saponification value (mg KOH/g)	240.590 ± 0.014	236.9 ± 2.7
Iodine value g (I_2_/100 g)	7.087 ± 0.006	18.3 ± 0.5
Peroxide value (meq/1000 g)	nd	nd
Acid value (meq/1000 g)	0.677 ± 0.006	0.592 ± 0.001
Ester value %	99.72 ± 0.01	99.41 ± 2.70

Nd: no peroxide was detected.

**Table 2:** ^1^H NMR data of indaiá and babassu oils in CDCl_3_.

Hydrogen nature	*δ*_H_ (indaiá oil)	*δ*_H_ (babaçu oil)
CH=CH	5.35	5.34
CH–O	5.24	5.26
CH–O	4.28	4.31
CH–O	4.14	**4.27**
CH_2_–CO	2.31	2.31
CH_2_–C=C	2.01	2.01
CH_2_–CH_2_–CO	1.6	1.6
CH_2_	1.25	1.25
CH_3_	0.86	0.87

**Table 3:** Fatty acid composition (mean percentage) of indaiá and babassu oils.

Fatty acid	Indaiá oil		Babassu oil	
	% Fatty acids	Retention time (min)	% Fatty acid	Retention time (min)
Caproic acid, C_6_	3.5	7.096	3.3	7.088
Caprylic acid, C_8_	10.9	7.998	9.2	7.986
Capric acid, C_10_	8.5	9.340	9.6	9.322
Lauric acid, C_12_	55.8	11.277	54.7	11.256
Myristic acid, C_14_	11.6	13.655	11.8	13.632
Palmitic acid, C_16_	3.8	16.234	4.8	16.207
Linoleic acid, C_18: 2_	nd	nd	0.9	18.779
Oleic acid, C_18:1_	5.8	19.569	6.5	19.547
Stearic acid, C_18:0_	nd	nd	2.05	20.678

Total unsaturated	5.8	—	9.45	—

**Table 4:** Assignments of the FT-IR bands of indaiá and babassu oil spectra.

Region	Indaiá	Babaçu	
1	—	3005	(=C–H) stretching
2	2958	2957	CH_3_ asymmetry stretching
3	2924	2920	CH_2_ asymmetry stretching
4	2854	2855	CH_2_ symmetry stretching
5	1746	1746	C=O stretching
6	1162	1163	C–O stretching
7	724	721	CH_2_ rocking
